# Barriers and Enablers of Healthy Eating Among University Students in Oaxaca de Juarez: A Mixed-Methods Study

**DOI:** 10.3390/nu17071263

**Published:** 2025-04-03

**Authors:** Patricia Jurado-Gonzalez, Sabina López-Toledo, Anna Bach-Faig, Francesc-Xavier Medina

**Affiliations:** 1 NUTRALiSS, Faculty of Health Sciences, Open University of Catalonia (UOC), 08018 Barcelona, Spain; pjuradogo@uoc.edu; 2 Health Sciences and Disease Study Center, Faculty of Dentistry, Benito Juarez Autonomous University of Oaxaca (UABJO), Oaxaca de Juarez 68120, Oaxaca, Mexico; sabina.ltoledo@gmail.com; 3 UNESCO Chair on Food, Culture and Development, Open University of Catalonia (UOC), 08018 Barcelona, Spain; fxmedina@uoc.edu; 4 Epi4Health Research Group, Faculty of Health Sciences, Open University of Catalonia (UOC), 08018 Barcelona, Spain

**Keywords:** dietary patterns, social ecological model, social cognitive theory, ethnography, young adults

## Abstract

**Background/Objectives**: The transition to university life brings significant social, psychological, and environmental changes, making it a critical period for establishing long-term dietary habits. However, many Mexican university students fail to meet national dietary guidelines, increasing their risk of non-communicable diseases. This study examines the determinants of healthy eating among university students in Oaxaca using a holistic, multi-level approach grounded in the Social Ecological Model (SEM) and Social Cognitive Theory (SCT). **Methods**: A mixed-methods approach was employed, integrating ethnography with a validated self-report questionnaire completed by 411 students at the *Universidad Autónoma Benito Juárez de Oaxaca* (UABJO). The ethnographic data included observations, field notes, photographs, informal conversations, and 13 semi-structured interviews. Data triangulation provided a comprehensive understanding of dietary behaviors by capturing both self-reported patterns and real-world eating practices and the food environment, as captured through ethnographic methods. The analysis included descriptive statistics, normality tests, and parametric tests to assess significant differences. **Results**: The findings revealed a decline in diet quality, characterized by low fruit and vegetable intake, high snack consumption, and irregular meal patterns, particularly among students living independently. Key barriers included academic stress, time constraints, low cooking self-efficacy, limited nutritional knowledge, peer pressure, and negative social norms, which contributed to reliance on convenient, processed foods. The lack of healthy food options on campus and the high perceived cost of nutritious food further led students to prioritize cheap, calorie-dense foods over healthier choices. Conversely, enablers included structured university schedules; peer support; hands-on culinary interventions; and improved access to affordable, healthy food. **Conclusions**: Addressing these barriers requires multi-level interventions that enhance nutrition literacy, cooking self-efficacy, and peer-led strategies while improving the campus food environment. Future research should explore SCT-based initiatives leveraging social networks and culinary education to foster sustainable dietary behavior change in university settings and assess how these findings can be applied in other socioeconomic and cultural contexts.

## 1. Introduction

University entrance is a critical transition period characterized by significant changes in social, psychological, physical, and cognitive development, alongside shifts in relationships with the social and food environment [1]. During this stage, young adults begin to take greater responsibility for their food choices, seek autonomy, and form their own identities. These factors often lead to negative dietary patterns, increasing the risk of non-communicable diseases (NCDs) [2,3]. For instance, first-year university students experience an average annual weight gain of 3–4 pounds, significantly higher than the weight gain typically observed in adolescents and adults over their lifespans [2,3]. Moreover, health behaviors developed during this stage often persist into adulthood, making it a crucial window for early intervention to prevent NCDs [4]. As a result, the diet quality of college students has emerged as a pressing public health concern [5].

Within this context, many Mexican university students face challenges in maintaining a healthy diet [6]. According to the National Health and Nutrition Survey [7], 38% of adolescents aged 12 to 19 are overweight [8], and only 16–17% of children and adolescents meet the recommended intake of fruits and vegetables [9]. A study in Jalisco analyzing the diet quality of college students revealed that fewer than 30% of university students adhere to a healthy diet, characterized by the low consumption of fruits and vegetables and the high intake of meat, sugars, and alcohol [10]. Similarly, a study in Oaxaca, based on a broader sample that included both university students and other population groups, revealed a high intake of cereals, meat, and processed foods [11]. In addition to unbalanced diets, university students frequently exhibit unhealthy behaviors, such as prolonged fasting; irregular meal times; and frequent meal skipping, especially breakfast [10].

Analyzing the factors that shape students’ dietary behaviors is essential for designing targeted interventions that address their unique needs, motivations, and barriers [1,12]. This requires a comprehensive theoretical framework that considers both external influences and internal motivations. To achieve this, this research applies the Social Ecological Model (SEM) to provide a multi-dimensional approach for understanding individual-, interpersonal-, community-, and policy-level influences, alongside the Social Cognitive Theory (SCT) to explore the psychological and motivational determinants of dietary behavior.

The SEM offers a comprehensive framework for examining health behaviors within a broader socio-ecological context, encompassing individual beliefs, interpersonal relationships, community policies, and environmental factors [13]. This model recognizes that food choices are shaped by the context in which they are made [13]. The SEM consists of four interrelated components: the individual level, which includes personal beliefs, attitudes, psychological factors, and demographic characteristics; the interpersonal level, which focuses on the dynamics of relationships among individuals, such as family and friends; the community level, which includes broader infrastructures that shape health-promoting behaviors, like schools; and, finally, the outermost layer, the policy level, which addresses food policies or regulations influencing food access [14].

Research highlights several factors influencing university students’ dietary behaviors [14,15,16,17,18]. At the individual level, psychological factors such as perceived stress, insufficient cognitive resources, anxiety, sadness, and low levels of food regulation contribute to unhealthy eating behaviors [17,19]. Emotional eating, body dissatisfaction, self-weighing, and a period of rebellious behavior further complicate food choices during this life stage [4,19,20]. These mental health challenges not only heighten the perception of barriers to healthy eating but also diminish students’ motivation to adopt healthier dietary behaviors [19]. Other individual barriers from the literature include limited time, lack of motivation for healthy eating, lack of culinary and nutritional knowledge, high screen usage, and a sedentary lifestyle [18,21]. Socioeconomic status, particularly parental education and household income, is another relevant individual barrier which impacts diet quality, as higher-income families tend to have better access to nutritious foods [9,22,23]. At the interpersonal level, the influence of peers and roommates to conform to unhealthy eating habits has been proven to play a crucial role, as social interactions and shared values around food impact choices [18,24]. In the physical environment, university food settings often provide easy access to processed and unhealthy foods while offering limited healthier options [4,25].

At a broader societal level, cultural norms and social media exposure, especially through influencers and marketing tactics, promote the consumption of energy-dense, nutrient-poor foods [26,27,28]. Finally, at the policy level, the high cost of healthy foods remains a significant structural barrier to better food choices [18].

While these barriers are well documented globally, there is limited research on the key determinants of healthy dietary behaviors among university students in Oaxaca, Mexico. Most studies in the region focus on adult dietary patterns or general diet quality, with little attention to university students or the application of robust theoretical frameworks [10,22,29]. Oaxaca’s cultural diversity, shaped by a rich blend of food traditions, high migration levels, and the presence of more than 19 distinct ethnic groups with varying cultural and socioeconomic contexts [30,31], further underscores the need for localized research, as findings from other regions may not be directly translatable.

Additionally, this research is grounded in Social Cognitive Theory (SCT) to provide a more comprehensive understanding of the factors influencing dietary behaviors [32] and to guide intervention strategies for behavior change. Developed by Albert Bandura, SCT highlights the significance of reciprocal determinism, which describes the interplay between personal factors and the socio-environmental context in shaping behavior [33,34]. It also emphasizes self-efficacy, an individual’s belief in their ability to successfully perform a behavior. Furthermore, SCT incorporates positive reinforcement, observational learning, and outcome expectations, all of which play a key role in facilitating long-term behavior change [34,35]. By integrating these principles, SCT adds depth to the individual and interpersonal levels of SEM, helping to explain the determinants of student eating habits and behaviors, as well as how these can be leveraged for intervention design.

By combining these theoretical perspectives, this research aims to explore the complexities of student eating behaviors and identify the motivators and barriers affecting Mexican university students in Oaxaca de Juárez in their ability to maintain a healthy diet. The findings will inform the development of context-specific, theory-driven interventions tailored to the unique needs, challenges, and drivers of dietary choices among university students, ultimately contributing to more effective health promotion strategies in Oaxacan academic settings.

## 2. Materials and Methods

### 2.1. Study Design and Participants

**Figure 1:**
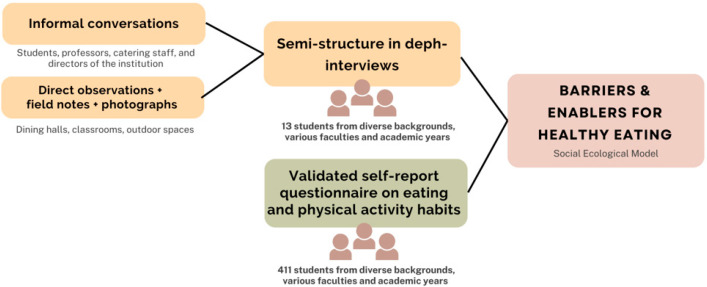
Framework for analyzing diet quality and the determinants of healthy eating among university students at UABJO.

This mixed-methods study employed a focused ethnographic approach to explore the socio-ecological contexts influencing dietary habits and a validated self-report questionnaire assessing eating and physical activity habits [36,37]. Figure 1 illustrates the research framework for analyzing barriers to healthy eating. Data were collected at the UABJO between November and December 2024.

Eligible participants for both the semi-structured interviews and questionnaire were university students enrolled in various programs at UABJO. The inclusion criteria were (1) being 18–22 years old and (2) enrollment in UABJO faculties in Oaxaca de Juárez. The exclusion criteria included (1) previous culinary education, (2) non-Mexican citizens, and (3) enrollment in distance education degree programs. Participation was voluntary, with no financial compensation. The participants had no prior relationship with the interviewers.

### 2.2. Recruitment

A multi-channel approach was used to recruit participants, combining snowball sampling with direct outreach methods to ensure diverse representation.

Initial recruitment involved emails sent through department deans at UABJO, encouraging participation from students across various academic fields. The email provided information on the study’s objectives, voluntary participation, and eligibility criteria.

Participants were screened based on the inclusion criteria and their sociodemographic characteristics, including sex, to ensure a gender-sensitive and diverse sample. Students from various faculties and academic years were selected to enhance representativeness.

Following initial recruitment, the snowball sampling method was employed to further extend participant outreach [3,38,39]. Early participants were asked to recommend peers who met the study criteria, helping to reach additional students who might not have been directly contacted through initial recruitment efforts. This method facilitated a broader, interconnected sample, offering valuable insights into shared experiences and variations within social networks [40].

### 2.3. Study Procedures and Data Collection

#### 2.3.1. Ethnography Methods

Ethnography is a qualitative research approach used to understand the cultural context, behaviors, and practices in naturally occurring settings. It is widely used to learn about people and their cultural context [41]. Mainly rooted in anthropology, but also in sociology, it usually gathers empirical insights about social practices to understand everyday behaviors [42]. In particular, ethnography has been widely used as an in-depth qualitative way to understand the complexity of health behaviors and its contributing factors, as they are highly influenced by the sociocultural ecological environment [37,43]. Food choices are inherently shaped by social, cultural, political, historical, and economic realities [13]. Therefore, ethnography is a useful method to understand mental models, attitudes, and social norms influencing food behaviors [44,45]. Focused ethnography was selected over traditional ethnography due to the limited study timeframe, more defined research questions, and emphasis on applied outcomes [46].

The ethnographic methods included direct observations, field notes, digital photographs, informal conversations, and semi-structured interviews [47]. Table 1 provides a summary of the ethnographic methods used in this study.

##### Direct Observations, Field Notes, and Digital Photographs

Direct observations were conducted in various university settings, such as dining halls, classrooms, and outdoor spaces, to capture the social and cultural context of eating habits [48]. A field diary documented reflections and key findings, while digital photographs enriched the field notes by visually contextualizing the food environment [49]. Privacy considerations were carefully maintained throughout.

##### Informal Conversations with Participants in Natural Settings

Informal conversations with various actors in the university environment were facilitated to supplement field research. These discussions served as initial points of contact and helped inform the design of the semi-structured interview questions [50]. Informal conversations occurred naturally in various settings. These dialogues were closer to everyday conversations than formal interviews [51]. These included observed conversations during fieldwork, such as sitting in the dining hall and noting student interactions, as well as participatory conversations involving direct dialogue between the researcher and students [52]. The key actors included students, professors, catering staff, and directors of the institution. These individuals were able to provide insights across the individual, social, and physical levels, enriching the analysis. Key insights were recorded in the field diary, as these conversations were not audio-recorded.

##### Semi-Structured Interviews

Thirteen semi-structured interviews were conducted as the primary qualitative data source, balancing systematic data collection with the flexibility to explore participants’ experiences in depth [53]. The semi-structured interviews were conducted in December 2024. The interviews were guided by an initial script (Supplementary Material S1) based on the study objectives, prior observations, informal conversations, and the related literature [26,54,55,56,57,58,59]. The questions followed the SEM, addressing barriers to and enablers of healthy eating across multiple levels of influence.

Semi-structured interviews were preferred over focus groups to elicit detailed, personal narratives about motivations for and barriers to healthy eating, avoiding the group dynamics that may influence disclosure in focus groups [60,61].

The interviews lasted around 20 to 40 min. A comfortable and quiet place was selected for the interviews in each of the faculties. Semi-structured interviews continued until data saturation was reached, meaning no new themes emerged. All interviews were conducted by the principal investigator, who facilitated the discussions and encouraged participants to speak freely and share their perspectives. The moderator did not express or give personal opinions verbally or nonverbally. Before each interview, participants provided written consent for audio recording and data processing.

#### 2.3.2. Validated Self-Report Questionnaire on Eating and Physical Activity Habits

A validated self-report questionnaire on eating and physical activity habits among 2710 Mexican adolescents developed by Martínez Coronado et al. (2023) [8] was adapted and distributed online to 411 students. The 28-item questionnaire covered food consumption frequency, types of food, meal settings, and lifestyle factors. Three questions related to physical activity were excluded as they fell outside the scope of this study. Only the initial question, which inquired about general engagement in physical activity, was retained for contextual purposes. Questions regarding physical activity frequency and lifestyle were omitted. The participants reviewed and signed an informed consent form before completing the questionnaire.

Data triangulation, achieved through multiple data collection methods, enhanced the depth and detail of the findings, offering a holistic perspective on the complex factors influencing food behaviors [62].

This study was approved by the Research Ethics Committee of the Universitat Oberta de Catalunya (CE23-TE04).

### 2.4. Data Analysis

Descriptive statistics were used to present the sample characteristics [63]. Quantitative data from the questionnaire were analyzed using SPSS statistical software (Version 21; IBM Corp., Armonk, NY, USA) with a 95% confidence level (*p* < 0.05). Initially, descriptive analyses were conducted to summarize the key characteristics of the study variables, including calculating means, standard deviations, frequencies, and percentages to provide an overview of the data.

Given the sample size (N > 50), normality was assessed using the Kolmogorov–Smirnov test, considering data as normally distributed when *p* > 0.05. Subsequently, inferential statistical analyses were performed to identify significant differences between relevant variables, such as gender, living arrangements, and socioeconomic level. For normally distributed variables, an Analysis of Variance (ANOVA) was used to compare means. For non-normally distributed variables, the Kruskal–Wallis test was applied. This approach enabled a comprehensive exploration of the data, providing both a detailed descriptive overview and a rigorous inferential analysis to detect significant differences between groups.

The conversations from the semi-structured interviews were audio-recorded using Audacity^®^ 3.6.1 software (Version 3). The primary researcher transcribed the audio recordings using TurboScribe.ai 2025 software. The field notes and transcriptions were then analyzed with the software package ATLAS.ti^®^ (Version 25.0.1 (32922)) (Smit, 2002) [64]. A content analysis was conducted using an inductive thematic approach to identify key themes and patterns in the data. A thematic analysis was conducted through a systematic review of transcripts to identify recurring themes and main categories. The principal investigator re-read the notes and transcripts, highlighting relevant quotations to extract overarching themes. Codes were initially generated during a data analysis and iteratively refined through multiple rounds of review to ensure consistency and coherence. As new themes emerged, previously coded data were reassessed to establish patterns and relationships between the themes. The process involved constant comparison across data sources, allowing subcategories to be refined and consolidated into main categories that captured the key barriers to and enablers of healthy eating.

## 3. Results

This study presents the results from the integration of quantitative data and qualitative themes within a mixed-methods design to analyze the diet quality of college students in Oaxaca, along with the determinants influencing their dietary patterns. The integration of both approaches followed the principle of triangulation to ensure a comprehensive understanding of the topic. While the quantitative data shed light on students’ dietary patterns, the qualitative findings delve deeper into the underlying factors that shape their eating behaviors and decisions.

### 3.1. Participant Characteristics

This study included two distinct groups of participants: those who took part in the semi-structured interviews and those who completed the questionnaire.

#### 3.1.1. Semi-Structured Interview Participants

Thirteen participants were recruited for the semi-structured interviews, ranging in age from 18 to 22 years, with an average age of 19. Seven participants lived independently, while six resided with their families. The sample included nine women and four men, with the majority enrolled in Dentistry (*n* = 8), followed by Veterinary Medicine (*n* = 2), and one participant each from Medicine, Sports Science, and Art. The recruitment aimed to ensure diversity in academic backgrounds (science vs. non-science) and to facilitate comparisons between the eating habits of students living independently and those residing with their families.

#### 3.1.2. Questionnaire Participants

A total of 411 students from the UABJO, enrolled in faculties based in Oaxaca de Juárez, completed the questionnaire. The characteristics of the questionnaire sample are summarized in Table 2. The majority of participants were female (72%). Most students reported a low to middle socioeconomic status, with over 85% having a monthly income of less than MXN 10,000. Regarding living arrangements, 35% of students lived with their parents, 38% lived with friends or college peers, and only 18% lived alone. Participants were between 18 and 22 years old. However, due to a technical issue, the research team was unable to accurately capture the specific age distribution.

### 3.2. Dietary Patterns

In general, students reported a decline in their diet quality since starting university. “*Since I started college, I buy everything already prepared, literally everything. It’s usually hamburgers, sandwiches, sometimes chips, and occasionally something else*” (Male, 18 years).

Key dietary changes included reduced water consumption, fewer meals per day, skipped or irregular meal patterns, increased reliance on fast food, decreased intake of fruits and vegetables, and higher consumption of snacks.

#### 3.2.1. Key Staples Among University Students

Many students indicated that their diet mainly consisted of affordable and convenient staples such as rice, pasta, bread, and corn-based products. When eating out, meals often included meat, while at home, common protein sources were eggs, tuna, or grilled chicken or pork. Processed options, like ready-to-eat soups and canned beans, were frequently consumed. “*What I normally cook at home is the most basic—eggs, sandwiches, soup, rice, just the basics*” (Male, 20 years). Another student added: “*I eat to survive. The most common things I cook are grilled chicken breast, eggs, a salad, spaghetti, and of course, beans and rice*” (Male, 22 years).

#### 3.2.2. Vegetable Consumption Frequency

Vegetable consumption was notably low, with only 6.3% of students reporting daily intake, while 48% consumed vegetables just 0–2 days per week. Vegetable intake was significantly higher among students living with their parents compared to those living alone. Specifically, among students living with their parents, 30% consumed vegetables 0–2 days per week, while 18% consumed them 5–6 days per week. In contrast, 58% of students living alone consumed vegetables 0–2 days per week, with only 6% consuming vegetables 5–6 days per week. One student explained, “*Since I live alone, I don’t have anyone to cook for me. I know how to cook, but I just don’t have enough time. So, as I mentioned before, when I started college, I began eating more processed foods and stopped eating vegetables and fruits. I kind of put them aside*” (Male, 20 years). When students did consume vegetables, it was typically limited to occasional salads served with fried meat at the university or at home.

#### 3.2.3. Fruit Consumption Frequency

Fruit consumption was slightly higher than vegetable intake but remained limited, with fewer than 12% of students consuming fruit daily. Among students living alone, 41% reported consuming fruits 0–2 days per week, compared to 23% of those living with family. Table 3 presents the frequency of vegetable and fruit consumption based on living arrangements. Students living alone were significantly more likely to have a low fruit intake and less likely to consume fruits daily, suggesting that living with family may support a more consistent and healthier fruit consumption pattern. When students did consume fruit, they typically opted for convenient fruit options, such as pre-cut fruit from street vendors or portable items like bananas and apples. “*When I eat fruit, it’s usually mango that I can buy already cut at a stall here*” (Male, 20 years). “*I often bring chopped fruit, like banana or apple*” (Female, 18 years).

#### 3.2.4. Beverage Consumption

Only 16% of students met the recommendation of drinking seven or more glasses of water per day. Many reported drinking less water since starting university, often due to forgetfulness or lack of time. “*I think my diet changed for the worse when I started college because when I was in high school, I used to go to the gym, eat well, and drink a lot of water. But now, I hardly ever drink water at university*” (Male, 18 years). “*I should try to drink more water, for example, because now I just forget*” (Female 18, years).

Interestingly, students living alone were less likely to consume soft drinks than those living with families or roommates. Among students living alone, 24% reported avoiding soft drinks altogether, compared to 13% of those living with family and 11% of those living with roommates.

#### 3.2.5. Processed Food and Snack Consumption

Most students reported occasional consumption of processed foods, such as processed meats, fried snacks, or sweets, with over 50% consuming these items 1–2 days per week. However, this contrasts sharply with the ethnographic research, which revealed that many students heavily relied on snacks as meal replacements, particularly during class breaks. Fried chips emerged as a staple due to their easy availability on campus. This discrepancy highlights a potential underreporting of snack consumption in the survey data. “*What I eat most regularly for lunch are Sabritas (fried chips)*” (Female, 18 years). This discrepancy underscores the importance of using multiple data collection methods to gain a more comprehensive understanding of dietary behaviors. While surveys rely on self-reported data, which may be influenced by recall bias or social desirability, ethnographic observations capture behaviors in real-life contexts, often revealing patterns that might not be fully reflected in structured responses [44].

**Figure 2:**
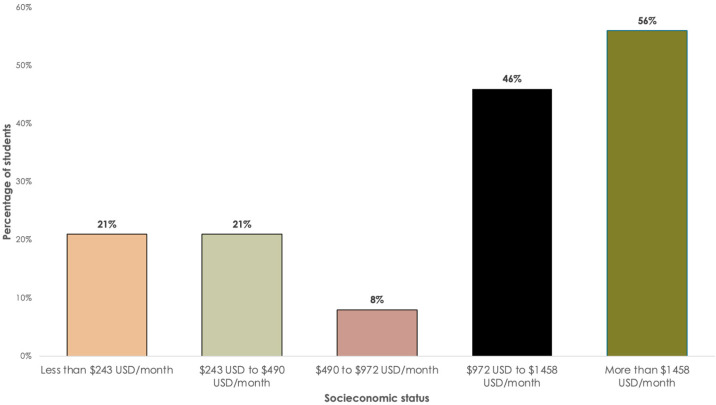
The percentage of students who consume snacks 3 to 4 days per week, categorized by their socioeconomic status.

Snack consumption patterns varied significantly by socioeconomic status (*p* = 0.048). Students from higher socioeconomic levels consumed snacks more frequently compared to those from middle- and low- income levels (see Figure 2). Additionally, students living with family or friends were more likely to consume snacks on campus (40% and 51%, respectively) than those living alone (26%).

#### 3.2.6. Dining Locations and Social Dining

The majority of students reported having their main meal—either lunch or breakfast—at home (48%) or at university (30%). Breakfast habits were strongly influenced by living arrangements. Students living with friends were more likely to have breakfast at university (48%) than at home (24%), while those living with family predominantly ate breakfast at home (42%).

Social dining patterns also differed by meal type. Breakfast was most often shared with friends (46%), whereas dinner was commonly eaten alone (46%) or with friends (39%). Students living with family overwhelmingly ate dinner at home (84%), while those living alone more frequently opted for street food or other external options.

#### 3.2.7. Physical Activity

A significant proportion of students did not engage in regular physical activity, with 38% reporting no exercise and 44% exercising occasionally. Gender differences were notable: men were more likely to exercise frequently, with 24% reporting very frequent activity compared to just 8% of women. Conversely, 41% of women reported rarely exercising, compared to 29% of men.

### 3.3. Determinants for Healthy Eating

**Figure 3:**
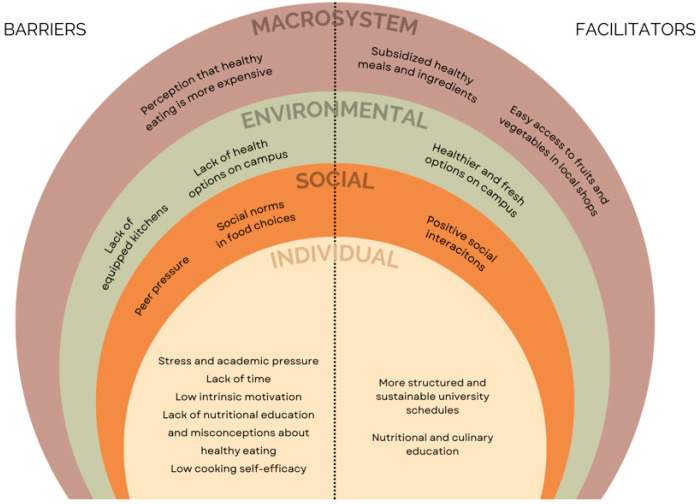
Summary of the barriers and enablers influencing healthy eating among university students, categorized according to the SEM: individual, social environmental, physical environmental, and macrosystem factors.

To better understand the factors influencing healthy eating among university students, Figure 3 summarizes the key barriers and enablers categorized according to the Social Ecological Model (SEM). These determinants are classified at different levels—the individual, social environmental, physical environmental, and macrosystem levels—highlighting the various challenges students face and the potential facilitators that could support healthier eating behaviors.

#### 3.3.1. Individual Factors

At the individual level, seven key determinants were identified, five barriers and two enablers. The main barriers included ‘stress and academic pressure’, which contributed to a perceived ‘lack of time’, as well as ‘low intrinsic motivation’, lack of ‘nutritional education and misconceptions about healthy eating’, and ‘low cooking self-efficacy’. The enablers included ‘more structured and sustainable university schedules’, as well as ‘nutritional and culinary education’.

##### Individual Barriers

Stress and academic pressure

Students reported that academic demands significantly impacted their food choices. Many described feeling overwhelmed, stressed, and exhausted, which led them to deprioritize basic needs like eating and sleeping: “*For example, right now that they’re starting to ask me for a lot of material, sometimes I have to prioritize the material over my food or my daily life*” (Male, 20 years). “*Stress significantly impacts my diet because even when there’s time, I feel too tired or stressed, and I lose the motivation to do anything, including eating*” (Female, 18 years).

During exam periods, this stress intensified, with students often skipping meals or consuming only one meal per day. “*During exams, I think the only meal I manage to have all day is one. The least I’ve eaten during those days is a sandwich*” (Male, 18 years). “*I arrive home really tired, and sometimes I end up sleeping instead of eating*” (Female, 18 years). One professor highlighted the consequences of prolonged fasting during exams, noting incidents of students fainting.

Many students coped with stress by consuming large amounts of caffeine, often substituting dinner with coffee and simple snacks like bread, cookies, or cereal.

Lack of time

Lack of time due to university commitments was the primary barrier identified during the ethnographic research. “*I don’t have time to cook. I make quick meals like salads, rice, or grilled chicken. Living alone means juggling cooking, tasks, and assignments*” (Female, 19 years).

Almost all participants mentioned that university schedules are usually intensive and last all day, leaving little time to eat. As a result, a common pattern is skipping meals, with most students eating only one or two large meals a day. “*I don’t have time to eat, and sometimes I don’t even manage to have three meals a day because of the time school takes up*” (Female, 22 years). Breakfast and dinner were particularly likely to be skipped. Survey data revealed that only 48% of students ate breakfast daily, and just 29% ate dinner daily, while nearly 20% reported never eating dinner. Significant gender differences were observed; 45% of men reported eating dinner daily, compared to 23% of women, while 20% of women reported never eating dinner, compared to 10% of men.

Breakfast and dinner habits also varied based on living arrangements. Students living with family were the most likely to eat breakfast daily (53%), followed by those living with roommates (49%), while students living alone were the most likely to skip breakfast (38%). Similarly, dinner was skipped more often by students living alone, with only 24% eating it daily, compared to 37% of students living with family.

Low intrinsic motivation

Many students expressed that even though they would like to eat healthily, it is not a priority during this period: “*I prefer to do homework rather than cook or eat*” (Female, 18 years). Students described feeling “lazy” about activities like grocery shopping, meal planning, and cooking. For some, this stemmed from not enjoying the cooking process. “*Fruits and vegetables are available where I live, but I’m too lazy to go and buy them*” (Male, 18 years) “*The main factors that influence me about cooking are time and laziness because I don’t enjoy it*” (Female, 18 years).

Lack of nutritional education and misconceptions about healthy eating

When asked about healthy diets, students revealed distorted perceptions of healthy eating, often paired with negative attitudes that heightened the barriers to adopting healthier habits. Healthy food was seen as unfulfilling, boring, lacking in flavor, expensive, and inconvenient. “*Healthy food doesn’t fill me up. For example, a plate of vegetables or a salad costs 60–70 pesos. For the same price, I could get a sandwich or tacos and feel full*” (Male, 20 years).

Low cooking self-efficacy

Students perceived healthy cooking as time-consuming and challenging. They struggle to plan, organize, and prepare healthy, quick, and convenient meals. “*When I started, I didn’t know how to organize my time or meals, so I only ate once a day” (Male, 19 years). “I would cook more healthily if I had more ideas for how to cook faster*” (Female, 20 years).

Additionally, most students viewed certain healthy foods, such as fish, legumes (beyond basic beans), and vegetables, as too complicated or time-consuming to prepare. This perception discouraged them from incorporating these healthy foods into their meals. “*It’s not that I don’t like legumes, but they’re hard to prepare and take time. For example, lentils take time to be cooked, and I don’t really like spending my time preparing food*” (Female, 18 years).

As a result, many students relied on simple, familiar staples like pasta, rice, canned tuna, grilled meat, eggs, and pre-prepared soups, which they found quicker and easier to prepare. “*I mostly cook eggs, with variations like eggs with ham or sausage. My diet is basically eggs, meat, and soup*” (Female, 18 years). “*My cooking is basic and just to survive. I usually make the essentials: eggs, salad, spaghetti, beans, and rice*” (Male, 20 years).

##### Individual Enablers

More structured and sustainable university schedules

Students suggested that having more structured university schedules could help them better manage their time and maintain regular eating habits. They proposed schedules that limited classes to either mornings or afternoons, along with designated breaks for meals. “*It would be helpful to have designated breaks for eating. It would allow us to have more structure*” (Female, 22 years).

Nutritional and culinary education

Most students recognized that improved nutritional education and cooking skills could motivate healthier eating habits. They emphasized the need for practical tools, such as quick recipes and meal-planning strategies “*If I knew more recipes… I would definitely make them at home. If I could make snacks at home in a healthier way, I would bring them to school and wouldn’t need to buy processed foods*” (Female, 18 years). “*I feel like if I had a better understanding of how to cook more things, I would definitely cook more, and it would help me eat healthier*” (Male, 20 years).

Students also expressed that learning more about nutrition and cooking could help them overcome many of the barriers they face, such as time constraints and low self-efficacy.

#### 3.3.2. Environmental Social Factors

At the social environmental level, the influence of peers, social interactions, and social norms around cooking and healthy eating emerged as both barriers to and enablers of healthy eating. Key barriers included ‘peer pressure’ and ‘social norms’, while ‘positive social interactions’ was identified as an enabler.

##### Environmental Social Barriers

Peer pressure

Students reported that peer dynamics played a significant role in shaping their food choices, often encouraging less healthy options. Dining with peers frequently resulted in eating fast food, as individuals tended to conform to the group’s choices. “*I think it’s less healthy because if one person orders something, we all tend to order the same thing because it appeals to everyone. So yes, it’s much less healthy. In fact, whatever we feel like, we buy it, and we all share it*” (Female, 18 years). “*Eating with peers highly influences me because sometimes they order burgers, and then I crave one too. Maybe I had planned on something else, but if someone orders something, it affects me, and I end up wanting it too*” (Female, 18 years).

In contrast, dining with family tended to encourage healthier habits, as family meals often included more traditional dishes with less grease and a higher content of fruits and vegetables.

Social norms in food choices

Fast food consumption was frequently identified as a dominant social norm during peer gatherings. Many students expressed that healthy eating was not highly valued within their social circles, making fast food the default option in group settings. “*If they order a hamburger, then I do too. It’s just what happens when we’re with friends—going out to eat burgers or getting tacos de barbacoa nearby*” (Male, 20 years). This social reinforcement of unhealthy eating behaviors reflects the strong influence of peer dynamics. Shared food choices and normalized behaviors perpetuate fast food consumption, further limiting opportunities for healthier alternatives.

##### Environmental Social Enablers

Positive social interactions

While peer influence often leads to unhealthy eating, it could also encourage healthier behaviors under the right circumstances. Some students reported eating more structured meals when dining with others, compared to the less deliberate choices they made when eating alone. “*I think when I eat with my friends, I tend to eat a bit, not healthier exactly, but maybe more structured than what I would prepare for myself*” (Female, 18 years).

Additionally, a few students noted that they would feel more motivated to eat healthily if their peers made similar choices. “*If my friends say, ‘Let’s go eat somewhere healthier’, I’d go. But if they suggest burgers, I’ll go with that. It’s all about what your friends decide*” (Male, 19 years).

Living or cooking with peers was also seen as an opportunity to make meal preparation more enjoyable and social. “*Cooking with friends would be more fun because I wouldn’t just enjoy the food myself, I know others would enjoy it too. If it’s just for me, I would do something really quick and easy*” (Female, 22 years).

#### 3.3.3. Physical Environmental Factors

At the physical environmental level, three key determinants were identified, two barriers—‘lack of healthy food options on campus’ and ‘lack of equipped kitchens’ in students’ apartments, particularly when living alone—and one enabler—‘healthier and fresher options on campus’.

##### Physical Environmental Barriers

Lack of healthy options on campus

Many students cited the limited availability of healthy food options on campus as a major barrier to maintaining a nutritious diet. Most food offerings were described as greasy, fried, and fast, with limited access to fresh or nutritious choices. “*For example, I’d say that the food at the university is very greasy. It tastes good, it’s cheap, but it’s unhealthy and all the same*” (Female, 18 years).

**Figure 4:**
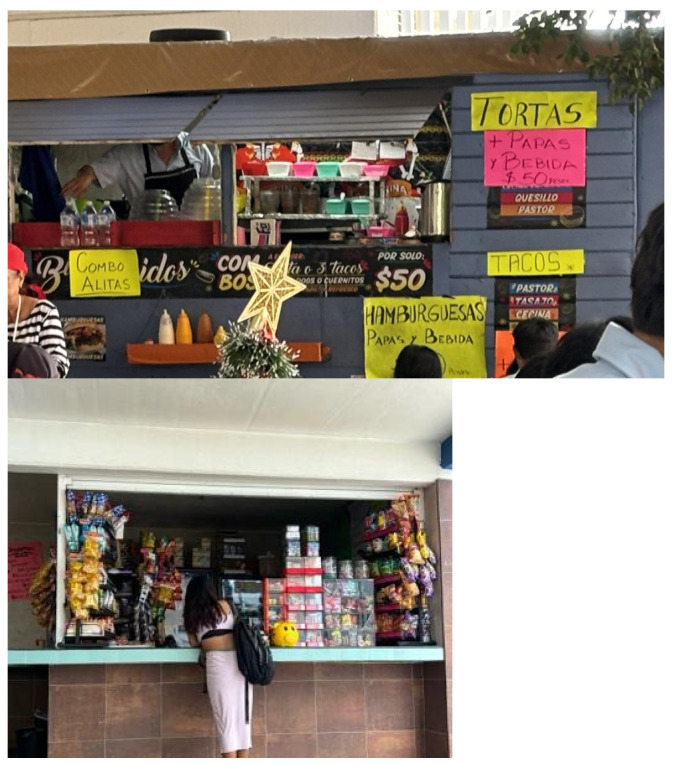
Example of a campus vendor at UABJO, reflecting the predominance of calorie-dense, processed foods and snacks in the campus food environment.

**Figure 5:**
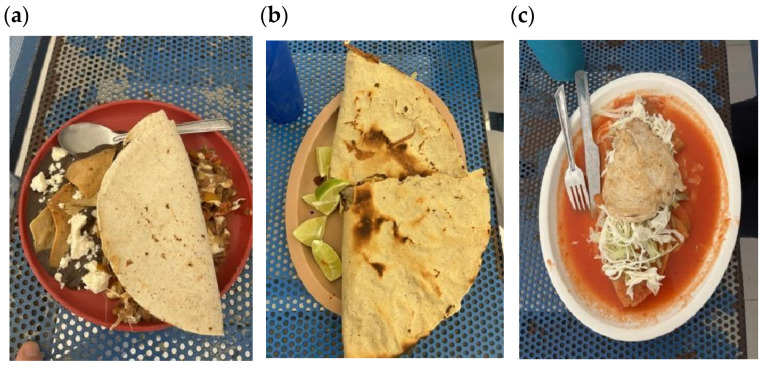

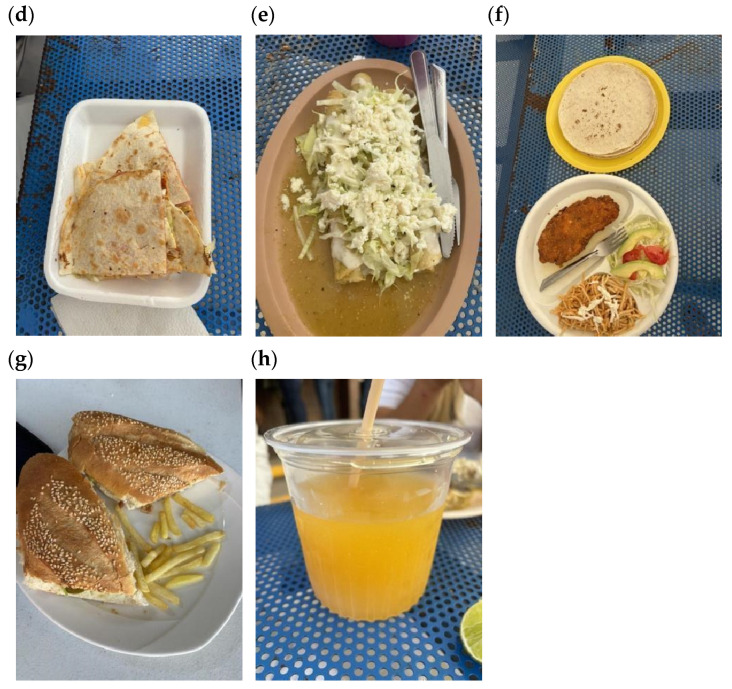
Examples of common lunch plates. (**a**) *chilaquiles with mole negro* (fried tortilla chips topped with a rich, dark mole sauce and sometimes with meat and sour cream); (**b**) *tlayudas* (a large, crispy tortilla topped with refried beans, cheese, cabbage, and grilled meat, often accompanied by salsa); (**c**) *enchiladas with meat* (rolled corn tortillas filled with meat (usually beef or chicken), covered in a red or green chili sauce, and baked with cheese); (**d**) *sincronizadas* (grilled tortillas filled with cheese and ham, and then lightly toasted); (**e**) *tacos dorados* (fried tacos filled with meat or potatoes, typically served with salsa, lettuce, and sour cream); (**f**) fried chicken; (**g**) meat sandwich typically served with lettuce, tomato, and mayonnaise on a soft bun; (**h**) orange sugary drink commonly served in large portions (0.5 L).

The food environment at campus kiosks was heavily dominated by sandwiches filled with meat and cheese, as well as tacos in various styles, often accompanied by sauce and processed meats, and fried meats. Fried snacks, such as potato chips, were also commonly offered, alongside instant noodles, which the vendor would prepare by adding hot water. In addition, sugary beverages were widely available, including horchata (a sweetened rice drink), fruit juices, flavored waters (especially popular among male students), and ice cream, or “nieves” as they are called in Mexico. Students noted that such offerings promoted excessive consumption of snacks, as healthier alternatives like fruits and vegetables were scarce (for more details on food vendors, see Figure 4, and common meals found on campus and consumed by students on their daily basis, see Figure 5). “*I wish there were more fruit at the university. They barely sell any fruit—there’s just one lady selling some, and that’s it*” (Male, 18 years).

Healthier food options tended to require more time to prepare, making them less convenient for students with busy schedules. Additionally, these healthier alternatives were often more expensive, creating financial barriers for many students.

Lack of equipped kitchens

Students who lived alone commonly cited inadequately equipped kitchens as a barrier to preparing healthy meals at home. Limited kitchen space, insufficient cooking appliances, and the inability to multitask while cooking were frequently mentioned. “*My kitchen doesn’t have much space for cooking. I can’t cook multiple things at once; I have to wait for one thing to finish before starting another. It takes a lot of time, honestly*” (Female, 18 years).

This lack of resources discouraged students from preparing meals, further limiting their ability to eat healthily at home.

##### Physical Environmental Enablers

Healthier and fresher options on campus

Students strongly expressed the need for accessible, quick, and appealing healthy food options on campus. They suggested including more fresh and balanced offerings, such as fruits, salads, soups, and meals with a balance of protein, vegetables, and carbohydrates. “*It would be nice to have more homestyle meals, like soups or dishes you’re used to eating at home. Right now, it feels like everything is just fast food*” (Female, 22 years).

Although some students admitted they might not consistently choose healthier options, they appreciated having the choice and the flexibility to balance fast food with healthier meals. “*I feel like I would choose healthier options, but not every day*” (Female, 18 years).

#### 3.3.4. Macrosystem Factors

At the macrosystem level, three key determinants were identified: one significant barrier, the perception that healthy eating is more expensive, and two enablers, subsidized healthy meals and easy access to affordable fruits and vegetables in local shops.

##### Macrosystem Barriers

Perception that healthy eating is more expensive

Students widely perceived healthy eating as expensive, both in terms of ingredient costs and meal preparation. They reported that cooking balanced and nutritious meals requires a larger budget compared to the affordability and convenience of fast food. “*I think the budget really affects it because buying ingredients for cooking is more expensive than buying something already prepared. For example, I don’t have many resources to cook, so for me, it’s a bit more expensive to cook at home than to eat something at the university*” (Male, 20 years).

Limited financial resources often led students to compromise on nutrition, opting for cheaper, less healthy options. “*Eating healthy is great, but economically not doable. I live alone, so I limit myself a lot. Right now, I eat very unhealthily, to be honest*” (Female, 18 years).

Students described unhealthy foods as the most affordable option when money was tight. “*With limited money, the only thing you can afford is unhealthy food*” (Female, 18 years).

##### Macrosystem Enablers

Subsidized healthy meals and fruits and vegetables

Affordable pricing for healthy meals and ingredients emerged as a key enabler of improved dietary habits. Proposals such as subsidized healthy meals at universities or discounts for fresh produce were seen as solutions to mitigate the financial burden: *“I feel like if I had a bigger budget, I would buy more ingredients to prepare meals. Sometimes they’re just too expensive, and I end up cooking simpler things. If I had more money, I’d invest in better meals for myself*” (Male, 20 years). “*For those of us who are from out of town, it would be great if they gave us a grant or something to buy healthy food. Sometimes the budget isn’t enough for everything*” (Female, 18 years).

Easy access to affordable fruits and vegetables in local shops

For some students, access to fruits and vegetables was not a challenge due to the availability of affordable options near their homes. However, additional barriers—such as lack of time, motivation, money, and organization—hindered their ability to purchase and consume these healthier options: “*It’s easy to find fruits and vegetables where I live, but I just don’t feel like going out to get them*” (Female, 18 years). Therefore, improving affordability and convenience for healthy food options—could encourage healthier eating behaviors among students.

## 4. Discussion

To our knowledge, this is the first mixed-methods study to explore the determinants of healthy eating, both barriers and enablers, among university students in Oaxaca, based on a sample of 411 participants. Our findings indicate that students’ diet quality does not meet national dietary guidelines [65], emphasizing the urgent need for targeted interventions. The determinants of dietary behavior reflect a complex interplay of individual-, environmental-, and policy-level factors. Key barriers included time constraints from academic commitments, insufficient nutritional knowledge, low cooking self-efficacy, peer pressure, social norms reinforcing unhealthy eating, limited access to healthy food on campus, and the high cost of nutritious options. In contrast, enablers such as structured university schedules, peer support, and increased accessibility to affordable healthy food played an important role in fostering healthier dietary behaviors. These findings align with studies conducted in other contexts, suggesting that university students globally encounter similar challenges [1,15,26,66].

Our study supports prior evidence suggesting that transitioning to university life is often associated with a decline in diet quality, weight gain, irregular eating patterns, and poor dietary choices [67,68,69]. Students frequently reported a decline in fruit and vegetable intake, irregular meal patterns, and an increased reliance on energy-dense snacks and fast food [70]. Notably, high-fat, high-sugar snacks were particularly prevalent among students living with friends, where snacking was normalized within shared living environments. This pattern mirrors findings from other university settings, where high snack intake contributes to increased risks of obesity and metabolic syndrome [71]. However, a notable discrepancy emerged between survey responses and ethnographic observations concerning snack consumption. While the survey data suggested that most students consumed processed snacks only occasionally, ethnographic research revealed a high daily consumption. This discrepancy suggests that students may underreport their actual snack intake in surveys, potentially due to recall bias or the perception that snacks are less significant than main meals [72]. This discrepancy highlights the value of mixed-methods approaches in providing a more comprehensive and nuanced understanding of dietary behaviors [73,74], capturing both self-reported habits and real-world food choices observed in daily life [37,44]. In addition to high snack consumption, low water intake and high consumption of sweetened drinks and coffee were prevalent among participants, reflecting national trends in Mexico, where 86% of Mexican adolescents aged 12–19 reported daily sweetened drink consumption [7]. A significant number of students relied on coffee at night as a meal replacement, often accompanied by sugary snacks like cookies or cereals, a trend consistent with global patterns of increasing caffeine consumption among university students [75]. Excessive caffeine consumption has been associated with poorer sleep quality and higher stress levels [76,77], raising concerns about its broader implications for student health.

Among the most significant barriers to healthy eating, time constraints due to academic demands emerged as the most prevalent, consistent with findings from other studies [15,18,26,57]. The demanding nature of university life led students to adopt time-constrained eating behaviors, frequently resulting in skipped meals or reliance on quick, energy-dense snacks [19,26,78]. In this study, 63% of students did not adhere to regular meal schedules, with only 48% consuming breakfast daily and just 29% consistently eating dinner. Many students found it challenging to balance academic responsibilities with maintaining a healthy diet. Eating behaviors further worsened during exam periods, with some students consuming only one meal a day or relying on energy drinks to cope with stress. Alarmingly, professors reported instances of students fainting due to long periods of fasting. To address these challenges, students suggested institutional changes, such as structured class schedules that include designated meal breaks, ensuring students have sufficient time to maintain a balanced diet.

Beyond time constraints, low cooking self-efficacy and limited nutritional education also emerged as critical barriers to healthy eating. Many students perceived healthy meals as unfulfilling, expensive, or too time-consuming to prepare, leading them to opt for processed and pre-packaged foods [15]. These misconceptions, combined with low confidence in cooking skills, further reduced students’ motivation to prepare nutritious meals. Our findings suggest that students would be more inclined to eat healthily if they had the necessary knowledge and skills, emphasizing the importance of cooking self-efficacy in bridging the gap between knowledge and action [79,80]. Research has shown that higher cooking self-efficacy is strongly associated with healthier food choices (Pope et al., 2021) [81] and can be effectively enhanced through hands-on culinary interventions grounded in SCT [35]. Tailored interventions that include hands-on cooking experiences, interactive learning, social support, and efforts to dispel negative outcome expectations regarding healthier food choices, have proven successful in building confidence and improving dietary habits [12,82]. Furthermore, linking healthy eating to students’ personal interests and social contexts may increase engagement in home cooking and promote long-term and sustainable dietary changes [1,17,26,83].

Peer influence and social norms had a dual effect, functioning as both barriers to and potential enablers of healthier eating [15]. During their university years, students often use food choices as a form of self-expression [1], leading students to mimic the eating habits of their peers [84]. Many students reported feeling pressured to conform to their social circle’s dietary habits, fearing that making different food choices would set them apart [85]. This underscores the vulnerability of students during this transitional period, where the need for social belonging often takes precedence over individual health goals [1]. In this regard, our research found that eating with peers often discouraged healthy eating, as fast food was the norm within social circles. For instance, students living with friends consumed more snacks than those living alone, as snacking was normalized in shared environments.

However, peer support also emerged as a powerful enabler. Peer groups can serve as role models [1]. Students surrounded by health-conscious peers were more likely to adopt healthier eating habits to fit in and avoid social judgment [84]. Moreover, cooking with friends was described as a motivating and enjoyable experience, making students more likely to prepare and consume healthier meals [18]. In fact, SCT posits that social support from others, such as friends or family, serves as a precursor to self-efficacy and has been associated with improved nutrition behaviors [12]. These findings suggest that leveraging peer dynamics through peer-led culinary initiatives and social-based interventions could be an effective strategy for promoting healthy eating on campus [22,35,86].

Beyond peer influence, financial constraints further exacerbated unhealthy eating behaviors, particularly among students living independently. Many students perceived healthy eating as unaffordable, often prioritizing cheaper, calorie-dense foods over nutritious options [14]. Food insecurity remains a critical issue for many university students, with many struggling to afford healthy food options [14]. This highlights the need for policy-level interventions, such as subsidized healthy meals on campus, access to affordable fresh produce, and financial incentives for nutritious food choices.

Additionally, family and home food environments were consistently described as having a positive influence on students’ dietary patterns. Students who lived with their families were more likely to eat all three meals and had a higher intake of vegetables and fruits. Interviews further supported this finding, as many students reported eating better when dining with family, associating home-cooked and traditional meals with healthier dietary patterns. Family guidance also played a key role in helping students make healthier decisions, aligning with previous research highlighting the importance of family dynamics and support in shaping young adults’ food choices [56,87]. However, concerns have been raised about a decline in parental culinary skills, which may contribute to a lack of intergenerational transmission of cooking knowledge. This trend may help explain why many university students in our study reported low cooking self-efficacy [88].

The campus food environment played a significant role in shaping students’ dietary habits. The widespread availability of calorie-dense, processed foods—such as fried items, sugary beverages, processed meats, instant noodles, and packaged snacks—combined with the limited accessibility of fresh fruits, vegetables, and nutrient-dense options, created a setting that reinforced unhealthy eating norms. The lack of affordable and appealing healthy alternatives made it difficult for students to maintain a balanced diet, further normalizing the consumption of fast food and processed snacks. Despite these challenges, students expressed a strong willingness to choose healthier options if they were more accessible, affordable, and satisfying. The introduction of initiatives such as salad bars, meal subsidies, and a greater variety of nutritious grab-and-go options could significantly influence students’ food choices, aligning with findings from previous research [14,89]. However, the absence of such alternatives perpetuates a cycle in which unhealthy eating remains the default, making it increasingly difficult for students to adopt better dietary habits, even when motivated to do so. Moreover, the dominance of fast food in the campus dining landscape reinforces social norms around unhealthy eating, subtly pressuring students to conform to the prevailing food culture [90]. Addressing these environmental barriers by improving the availability, affordability, and appeal of nutritious meals could create a more supportive food environment, ultimately fostering healthier eating behaviors among university students.

Given the intersection of these determinants, addressing barriers to healthy eating requires a multi-sectoral approach. Research highlights the importance of focusing on modifiable environmental and psychological factors when designing behavior change interventions [1]. While factors such as academic stress and time constraints may be difficult to modify [91], interventions targeting nutrition and culinary education, as well as improvements to the campus food environment, are both feasible, urgent, and effective [1]. According to SCT, providing practical cooking education and nutritional literacy programs can enhance students’ confidence and motivation to prepare healthier meals, leading to sustained behavior change. Additionally, collaborating with campus food providers to create a supportive food environment—one that offers affordable, convenient, and nutritious options—could dispel misconceptions about the cost and convenience of healthy food.

Social dynamics also play a critical role in shaping food choices. Peer-based initiatives that promote communal cooking and reinforce healthy eating behaviors could shift social norms, making nutritious food choices more appealing and accessible. Given the influence of peer networks, leveraging social interactions as part of behavior change strategies could be a powerful tool for fostering healthier habits.

Based on these findings, effective interventions should adopt a holistic approach that integrates individual, social, and environmental factors, tailoring strategies to the specific context of Oaxacan students to foster healthier eating behaviors. By addressing these interconnected determinants, interventions can create sustainable dietary improvements that extend beyond university settings, ultimately supporting long-term health outcomes.

### Strengths and Limitations

On one side, this study has several limitations. First, there may be potential selection bias, as participation was voluntary, and recruitment was conducted through snowball sampling. It may have attracted students already interested in the topic of healthy eating. Additionally, the primary researcher’s non-Mexican background may have influenced data interpretation due to cultural differences. Efforts were made to mitigate this limitation using an anthropological methodology and the involvement of a local researcher. Moreover, the sample was predominantly female (72%), which limits the generalizability of the findings to the broader student population. Furthermore, due to technical issues with Qualtrics, the demographic question regarding students’ age or faculty did not appear in the survey, not allowing the collection of age-related data or faculty data for quantitative analysis.

On the other hand, this study possesses several strengths. The mixed-methods design, integrating ethnographic techniques with a quantitative survey, facilitated robust data triangulation, strengthening the reliability and depth of the findings [92]. The use of a validated survey tool to assess diet quality further strengthens the credibility of the results. Additionally, the sample size of students from the UABJO was statistically significant, offering a representative sample for the survey and providing valuable insights into the eating behaviors of university students in Oaxaca.

## 5. Conclusions

This study is the first mixed-methods investigation to apply the SEM and SCT frameworks in examining the determinants of healthy eating among university students in Oaxaca. Given Oaxaca’s distinct cultural and socioeconomic context, these findings reveal specific barriers and opportunities that shape university students’ dietary behaviors. Our findings indicate a significant decline in diet quality upon entering university, marked by reduced fruit and vegetable intake, increased consumption of snacks and caffeine, and irregular meal patterns. These trends were more pronounced among students living away from home, highlighting the challenges of maintaining a nutritious diet amid major life transitions, increased autonomy, and evolving social and environmental contexts during this developmental stage.

The key barriers identified, academic stress, time constraints, low cooking self-efficacy, limited nutrition knowledge, peer influence, social norms, and financial constraints, highlight the complex interplay between individual, social, and environmental factors shaping students’ dietary choices. Conversely, structured academic schedules; supportive peer networks; and increased accessibility to affordable, nutritious food emerged as critical enablers of healthier eating behaviors.

A critical contribution of this study is the identification of discrepancies between self-reported dietary behaviors and observed food choices through ethnographic methods. While survey responses suggested that students rarely consumed snacks and processed foods, ethnographic observations revealed a much higher reliance on these foods as meal replacements. This discrepancy underscores the limitations of self-reported dietary assessments and highlights the value of multi-method approaches in capturing both stated behaviors and real-world eating practices.

These findings align with research on university students in other countries, underscoring the importance of addressing socioecological factors that influence dietary behaviors. Enhancing cooking self-efficacy and nutrition literacy through culinary interventions, fostering peer support with peer-led approaches, and improving access to healthy food options on campus by leveraging local resources are promising strategies for promoting healthier eating behaviors among university students.

Future research should examine peer-led, theory-driven interventions and social support networks as strategies for promoting healthier eating behaviors. Additionally, hands-on culinary interventions incorporating SCT principles could be effective in fostering sustainable dietary changes. By integrating ethnographic insights with survey data, future studies can refine context-specific interventions to enhance student nutrition in Oaxaca, Mexico, and comparable settings. Further research should replicate this study with a larger, more gender-balanced sample to enhance generalizability.

## Figures and Tables

**Table 1:** Summary of ethnographic methods employed in the study.

Research Method	Objective
Direct observations	Explore and deepen the understanding of the cultural and social dynamics shaping eating behaviors within the university environment, with midday observations of how students interact among themselves and with food in the university setting to record their behaviors.
Field notes	Provide reflections on the research journey and establish a transparent record of the researcher’s experiences, enhancing the study’s scientific rigor.
Digital photographs	Enrich field notes by integrating visual depictions that illustrate the food environment and its characteristics.
Informal conversations	Serve as an initial method for gathering contextual insights into dietary habits and nutritional quality, engaging diverse stakeholders such as students, faculty, cafeteria staff, and institutional directors.
Semi-structured interviews	Act as a primary data source to identify barriers and facilitators for healthy eating.

**Table 2:** Summary of questionnaire respondents’ demographics. Overview of key demographic variables, including gender distribution, socioeconomic status, and living arrangements among survey respondents. Percentages reflect the proportion of participants within each category.

Variable	Category	Frequency (%)
Gender	Female	72%
	Male	26%
	Non-binary	1%
Socioeconomic status	Less than USD 243/month	54%
	USD 243 to USD 490/month	31%
	USD 490 to USD 972/month	9%
	USD 972 to USD 1458/month	3%
	More than USD 1458/month	2%
Living situation	Alone	18%
	Shared apartment with roommates	8%
	With family	35%
	With friends or peers	38%

**Table 3:** Frequency of vegetable and fruit consumption among university students based on their living arrangements. Percentages represent the proportion of students consuming vegetables and fruits 0–2 days/week, 3–4 days/week, 5–6 days/week, or daily across different living situations, including living with friends, with family, in a shared apartment, or alone.

	0–2 Days/Week		3–4 Days/Week		5–6 Days/Week		Daily	
	Vegetables	Fruits	Vegetables	Fruits	Vegetables	Fruits	Vegetables	Fruits
**With friends**	42%	29%	41%	39%	10%	30%	8%	12%
**With my family**	30%	23%	42%	45%	18%	17%	11%	15%
**In a shared apartment**	50%	21%	25%	44%	13%	22%	13%	13%
**Alone**	58%	41%	31%	45%	7%	9%	4%	4%

## Data Availability

Data will be made available on request.

## Supplementary Materials

The following supporting information can be downloaded at: https://www.mdpi.com/article/10.3390/nu17071263/s1.
