# Supplementary material for: Barriers and Enablers of Healthy Eating Among University Students in Oaxaca de Juarez: A Mixed-Methods Study
Source: Nutrients. 2025 Apr 3;17(7):1263. doi: 10.3390/nu17071263 (PMC11990523; doi:10.3390/nu17071263)
Supplement: Supplementary file 1 [file nutrients-17-01263-s001.zip › nutrients-3531550-supplementary.pdf]

Supplementary Material

Supplementary material S1: Interview script (translated from Spanish)
PART 1: ANALYSIS OF HEALTHY EATING FACTORS – 20 MINUTES
Individual
Nutrition-knowledge and beliefs

- Is “eating healthy” important to you?
- Describe a healthy college person.
- What does "eating healthy" mean to you?
- When you think about healthy eating for college students, what comes to mind?

Food self-efficacy

- If you decided to start eating healthier, what changes do you think you could easily make? What obstacles do you think you might encounter when trying to eat healthier?
- What kind of information or skills do you think you need to improve your diet?

Dietary changes since arriving at university

- How has the quality of your diet changed since you arrived at university?
    - What factors do you think have caused these changes?
    - Which of the factors you mentioned have had the greatest impact?
    - What do you think would be necessary to motivate you to eat healthier during your college days, or what strategies could help you overcome those barriers?
- How does stress or academic demands influence your dietary decisions?
- How do your cooking skills influence your food decision-making?

Motivation

- What is most important to you when deciding what to eat?
- Do you think you'd benefit personally if you ate healthier? What would you expect? (Better academic performance, more energy, etc.)?

Choosing places to eat

- What's your priority when choosing a place to eat at university? And when choosing what to eat?

Social environment

- How do you think social dynamics (eating with friends, going out in groups) influence the quality of your diet? That is, when you eat with friends, do you tend to eat more or less healthy? Why?
- How does what your friends think influence your dietary decisions?
- Do you think you would eat healthier if your friends or family did too?
- When you eat with your family, do you tend to eat more or less healthy? Why?

Physical environment

- How would you describe the food options available to you at the university?
- Do you think having healthier options at university would make it easier to eat healthier? What types of foods would you like to find more easily?
- Although there are small businesses near where you live, do you think there are enough healthy options available in your area?

Macrosystem

- If there was a place that offered you healthy preparations, even though it cost more ($) than the others, would you honestly choose it? Why?
- If the cost were the same as other meals, would you honestly choose it? Why?
- Do you think eating healthy is affordable for you?

PART 2: ANALYSIS OF FACTORS FOR COOKING AT HOME – 20 MINUTES
Individual
Self-efficacy in the kitchen
- Do you consider yourself to have culinary skills?
- Do you feel you have the knowledge to cook healthy meals at home?
- Where do the recipes or techniques you use for cooking come from?
- **How confident do you feel when cooking without following a recipe?**
- How comfortable do you feel trying new or healthier recipes?

Barriers and motivators
- What factors most influence your decision to cook at home (e.g., lack of time, lack of knowledge, lack of interest)?

Motivation to cook at home:
- When cooking at home, what is most important to you—health, price, flavor, speed…?
- What recipes do you cook most often at home and why?
- What would encourage you to cook more often (resources, support, planning)? And to cook healthier?

Barriers and motivators for cooking at home:
- What types of foods do you find the most difficult to cook, and that's why you don't usually cook them?
- What are the main barriers you face when trying to cook something more elaborate or healthy?

Social
Do you prefer to cook alone or with friends or family? Why?
Do you think cooking with friends or family would make cooking more fun or easier?

Physical
Do you think your kitchen is sufficiently equipped to cook at home?
If you had access to more resources (such as utensils or ingredients), do you think you would cook more?

Macro
How does your budget affect how often you cook at home?
